# Cancer Patients who Declined a Clinical Trial: A Pilot Study of Patient-Identified Barriers

**DOI:** 10.1188/21.CJON.647-654

**Published:** 2021-12-01

**Authors:** Mishellene McKinney, Rose Bell, Cindy Samborski, Kristopher Attwood, Grace Dean, Katherine Eakle, Wei Yu, Nessa Stefaniak, Stephen B. Edge

**Affiliations:** Roswell Park Comprehensive Cancer Center in Buffalo, NY; Roswell Park Care Network in Buffalo, NY; Roswell Park Comprehensive Cancer Center in Buffalo, NY; Roswell Park Comprehensive Cancer Center in Buffalo, NY; University at Buffalo in Buffalo, NY; Genentech, Inc., South San Francisco, CA.; Genentech, Inc., South San Francisco, CA; Roswell Park Comprehensive Cancer Center in Buffalo, NY; Roswell Park Comprehensive Cancer Center in Buffalo, NY

## Abstract

**Background:**

Little progress has been made in the past decade on improving clinical trial enrollment in the United States, particularly for adults and those in ethnic and racial minorities. Oncology nurses play a pivotal role in identifying and addressing patient concerns about clinical trials.

**Objectives:**

The aim was to identify patient-related barriers to clinical trial participation using a mixed-method patient survey and offer insights to develop evidence-based implementation strategies to address these barriers.

**Methods:**

A retrospective survey was conducted of patients who were not interested in participating in a clinical trial to quantify the reasons these patients chose not to participate. Directed qualitative content analysis was used to identify themes that emerged from the write-in responses.

**Findings:**

The greatest patient-reported barriers were misperceptions about placebos, not wanting to feel like a ‘human guinea pig’, uncertainty surrounding clinical trial treatment effectiveness compared to standard care, and concerns about additional appointments or tests. Oncology nurses can address patient enrollment barriers by providing targeted education and participating in the informed consent process.

## Background

Progress in cancer care requires ongoing clinical trials. Only 3–5% (Hallquist Viale, 2016) of adult cancer treatment in the United States is provided within a clinical trial (CT). Little progress has been made in the past decade on improving enrollment in the United States, particularly for older adults and those in ethnic and racial minorities(Sedrak et al., 2021). Barriers to cancer trial enrollment have been explored and reported extensively in the literature. The most common obstacles have been broadly categorized as structural, clinical, physician or patient-related (Lara et al., 2001; Mills et al., 2006; Nipp, Hong, & Paskett, 2019; Sedrak et al., 2021; Unger, Vaidya, Hershman, Minasian, & Fleury, 2019). Hillyer et al. report that there remains a wide disparity in provider versus patient attitudes and beliefs regarding clinical trials (2020). Oncology nurses play a pivotal role in identifying and addressing patient concerns about clinical trials.

### Structural Factors

In a systematic review and meta-analysis, structural barriers and clinical barriers accounted for more than 77% of patients not enrolling in a clinical trial (Unger et al., 2019). Structural barriers include the absence of an available trial at an institution and other factors such as limited clinical research staff support. The institution may not offer clinical trials at all or may not offer a trial that is appropriate for the patient’s cancer type or stage. Over 85% of patients in the United States receive their cancer care in a community setting, where there are fewer opportunities for trial participation than at an urban academic center (Network, 2021).

### Clinical Factors

Clinical barriers include restrictive clinical trial eligibility criteria. The intent of trial eligibility criteria is to assure a defined population to address the research question and to protect the safety of trial participants due to the potential impact the study may have on patients with more serious health issues. Despite recommendations from the American Society of Clinical Oncology to update trial eligibility to be more representative of the cancer patient population’s health, stringent eligibility criteria continue (Kim et al., 2017). Ineligibility rates in the United States are between 18.5% to 25.4% [. In particular, the expansion of personalized medicine (the use of genetic or other biomarker information to make treatment decisions about patients) has led to an increase in biomarker specific trials that limit eligibility to a small group of patients (Janiaud, Serghiou, & Ioannidis, 2019).

### Physician Factors

Physicians play a critical role in presenting trials to patients and helping them to understand the role of a clinical trial in their treatment. When eligible patients are presented with a trial by their physician, they agree to participate more than 50% of the time (Unger et al., 2019). Physicians may decide not to discuss trials with patients because of time constraints or treatment preference. They may also be unaware of trial options, or have concerns with the complex nature of protocols (Mills et al., 2006).This may be an area where oncology nurses can help patients through assisting in identifying trial candidates and educating patients about the value of clinical trials.

### Patient Factors

Patient-related barriers can include personal factors and beliefs that affect patient willingness to participate. Other factors identified include concerns of a negative impact on their relationship with their (Mills et al., 2006), patient and family dynamics (Hillyer et al., 2020), fear of placebo, loss of control, time required to participate, and fear of side effects (Hillyer et al., 2020; Mills et al., 2006; Nielsen & Berthelsen, 2019; Nipp et al., 2019; Sedrak et al., 2021).

This study focuses on patient-related factors where there are many actionable barriers to which oncology nurses can play a pivotal role in identifying and addressing.

## Purpose

The aim of this study was to 1) identify patient-related barriers to clinical trial participation using a mixed-method patient survey and 2) present oncology nurses with evidence-based strategies to address these barriers.

## Methods

### Sample and Setting

This study was a retrospective mixed-methods analysis of patients not interested in participating in a clinical trial. Quantitative analysis was used for closed ended survey questions and directed qualitative content analysis was used to identify themes that emerged from the write-in responses. The survey was conducted from January 24^th^, 2019 to June 30^th^, 2019. The Roswell Park (RP) Institutional Review Board approved this study and participants signed a written consent to participate in the survey.

Data from the point-of-care clinical oncology pathway (COP) system at RP Comprehensive Cancer Center in Buffalo, NY site was leveraged to help identify patients who were eligible for trials based upon cancer type, staging, relevant biomarkers and other clinical characteristics. In the COP, the medical oncology provider provides information on the cancer type, stage, biomarkers and clinical situation. If a clinical trial(s) is open for accrual at the center that matches the basic clinical situation, it is presented to the provider as the first treatment choice. The provider must then either select a trial or select from a list of reasons why a trial was not selected (e.g. patient eligibility, provider preference, insurance or cost, patient preference or “other reason”). If the provider selects the trial, the COP immediately sends an automatic message to the Clinical Research Coordinator to complete full eligibility screening for that trial.

### Offering a Clinical Trial

Clinical Research Coordinators (CRC) and Physicians at RP review a patient’s medical history to ascertain if basic eligibility is met. Physicians introduce the Standard of Care (SOC) and CT to the patient as treatment options. If the patient chooses CT, the CRC reviews the research study consent in depth to ensure the patient understands the trial. Once consent is obtained, study related tests are initiated to determine final eligibility.

### Patient Eligibility and Recruitment

The survey study population consisted of adult English and Spanish-speaking patients receiving a COP recommendation for a cancer type and circumstance (e.g. adjuvant therapy, metastatic/recurrent cancer) where a clinical trial was presented to the provider in the COP for consideration. Eligible patients were identified using data from the COP. The data were filtered to identify patients who declined to participate and who had a solid tumor type in the breast, gynecology, gastrointestinal, genitourinary, melanoma, head and neck, and thoracic services.

If the provider documented in the COP, “Patient Not Interested in Any Trial” or “Patient Not Interested in This Trial”, and the patient met the other basic eligibility criteria for the survey a research staff member contacted the patient to join this study. The researcher also verbally confirmed with patients that they were not interested in participating in a trial. Eligible patients were consented for this study and a paper survey was given to the patient to complete and collected immediately.

### Data Collection Instrument

The mixed methods survey was based on a questionnaire used in a similar study at the Royal Marsden Hospital in London, England (Moorcraft et al., 2016). The questionnaire, licensed under the Creative Commons Attribution 4.0 International License with unrestricted permissions, was developed based on a review of the literature and the authors’ experiences of trial recruitment. The modified, mixed methods survey used for this study (Table 3) was expanded to include write in responses to questions 26–28 for richer qualitative analysis. The survey includes Likert (n=6), multiple choice (n=19),open-ended responses (n=3), and demographic questions. A panel of experts in oncology research (SBE, KE), and nursing research (MM, RB, GD) reviewed the modified version for face and content validity. The use of the opened ended survey responses and their analysis were intended *a priori* as an adjunct analysis to the primary survey research, with the intention of enhancing the analysis of closed-ended survey responses.

Survey study data were collected and managed using REDCap (Research Electronic Data Capture) tools hosted at RP Comprehensive Cancer Center. REDCap is a secure, web-based software platform designed to support data capture for research studies (Harris et al., 2019).

### Mixed Methods Analysis

A mixed methods approach was used to analyze the survey data. Statistical analysis was performed to determine the mean and range for continuous responses and counts and percentages for categorical response. Directed qualitative content analysis was used to identify concepts and themes that emerged from the three open-ended responses. The directed qualitative content analysis approach is generally used to describe a phenomenon that would benefit from further description (Assarroudi, Heshmati Nabavi, Armat, Ebadi, & Vaismoradi, 2018) and was used here to probe patients’ perceptions of participating in a clinical trial. The aforementioned expert panel coded the data and reached consensus about the final themes that emerged from the data.

## Results

### Trials Presented for Pre-Screening

During the study time period, there were 272 instances of trials offered to 164 unique patients that were categorized in the COP as “patient not interested”. Twenty-three of the patients were deceased before being approached about the survey and 75 were determined to be ineligible because the patient did not recall being offered a trial when approached by the researcher, or the patient was hospitalized or too ill to approach. 36 did not have a scheduled appointment at the cancer center within the enrollment window or were missed. Of the 30 patients that were approached to take the survey, 9 declined and 21 completed the survey, for a 70% participation rate.

### Quantitative Survey Data

Demographics and clinical characteristics can be found in Table 1. Most survey participants (81%; n=17) had a COP treatment decision for metastatic solid tumors. Most participants were female (66%; n=14) and non-Hispanic Caucasian (81%; n=17).. Most patients reported their education levels as college or higher (66%; n=14). The majority of patients had not participated in a clinical trial before (85%, n=17).

Travel time to get to the Institute varied. 53% (n=11) of patients stated it took more than 30mins to reach the cancer center with 29% (n=6) taking 1–2hrs. 71% (n=15) had someone else drive them (friend, family member or public transportation service)

Patients reported receiving the most information about trials from their oncologist (43%, n=9) or the CRC oncologist (43%, n=9). The family and friends that they reported discussing treatment with most were spouses (10) and their children (9).

Most patients surveyed (76%; n=16) saw being asked to participate in cancer research as a “positive thing”. No patients felt that it was a “negative thing” with the remainder of respondents (23%, n=5) seeing it as “neither positive nor negative”. Most responded positively (agree or strongly agree) to the statement “I believe clinical trials associated with cancer research will help doctors better understand and treat cancer” (90%; n=19). A few (10%; n=2) were concerned about the use and storage of blood and tissue samples for research. 1 patient (5%) reported being concerned about incurring additional costs because of trial participation. Overall, patients reported that the amount of information and time spent discussing the trial were adequate (Table 2). The most reported barriers in the multiple-choice response section were concerns about receiving a placebo (52%; n=11) and not wanting to feel like a ‘human guinea pig’ (43%; n=9).

### Qualitative Survey Data

When asked, “What would make you more interested in participating in a clinical trial?” 47% of patients wanted more supporting evidence for the trial, indicating a perceived risk about the quality of trial outcomes compared to standard of care outcomes. Response themes for the question, “Please explain the reasons that you decided not to enroll in a clinical trial,” indicated concerns about additional appointments or tests (28%) and perceived risk of participating in a trial versus standard care, including concerns of receiving a placebo and the uncertainty of the effectiveness of the treatment. When asked to explain what a clinical trial was in their own words, 14% of patient responses included the word “placebo.” There was no correlation between the word “placebo” appearing in the trial name offered and patients who voiced concern about receiving placebos in write-in responses.

**Figure 1:**
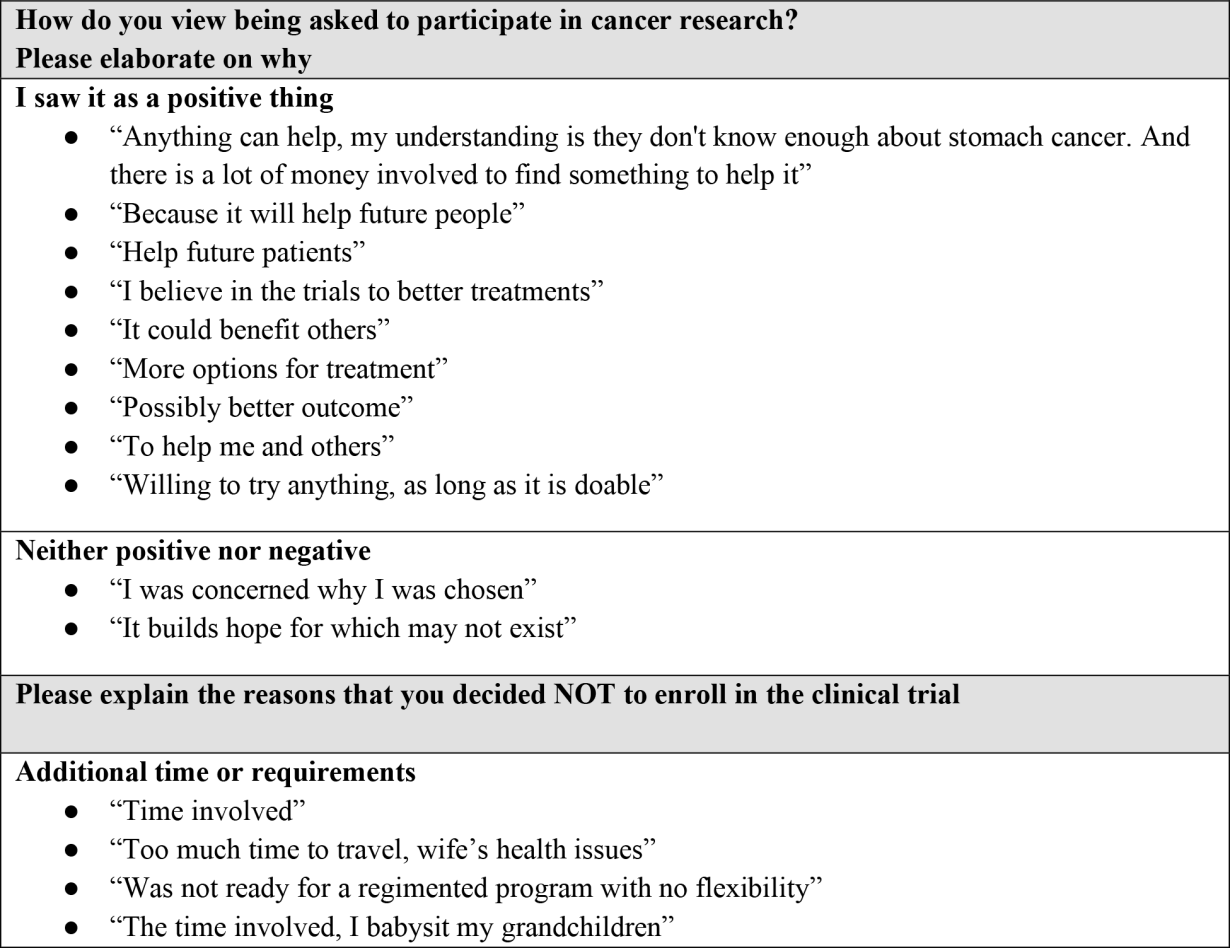

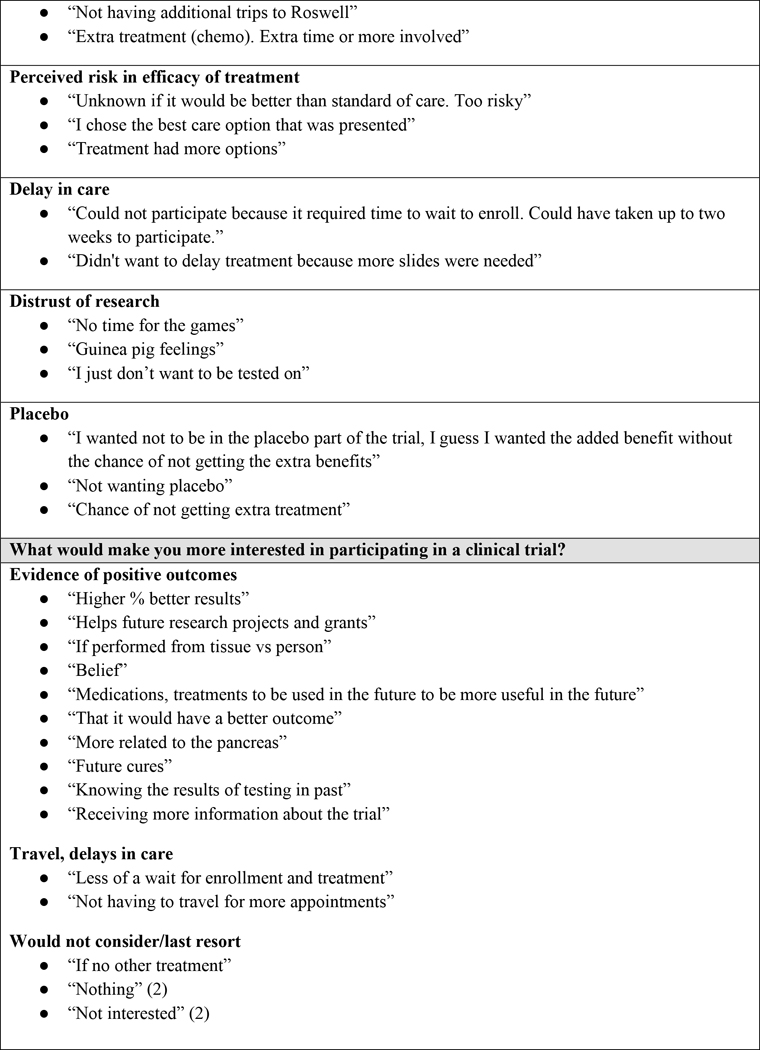

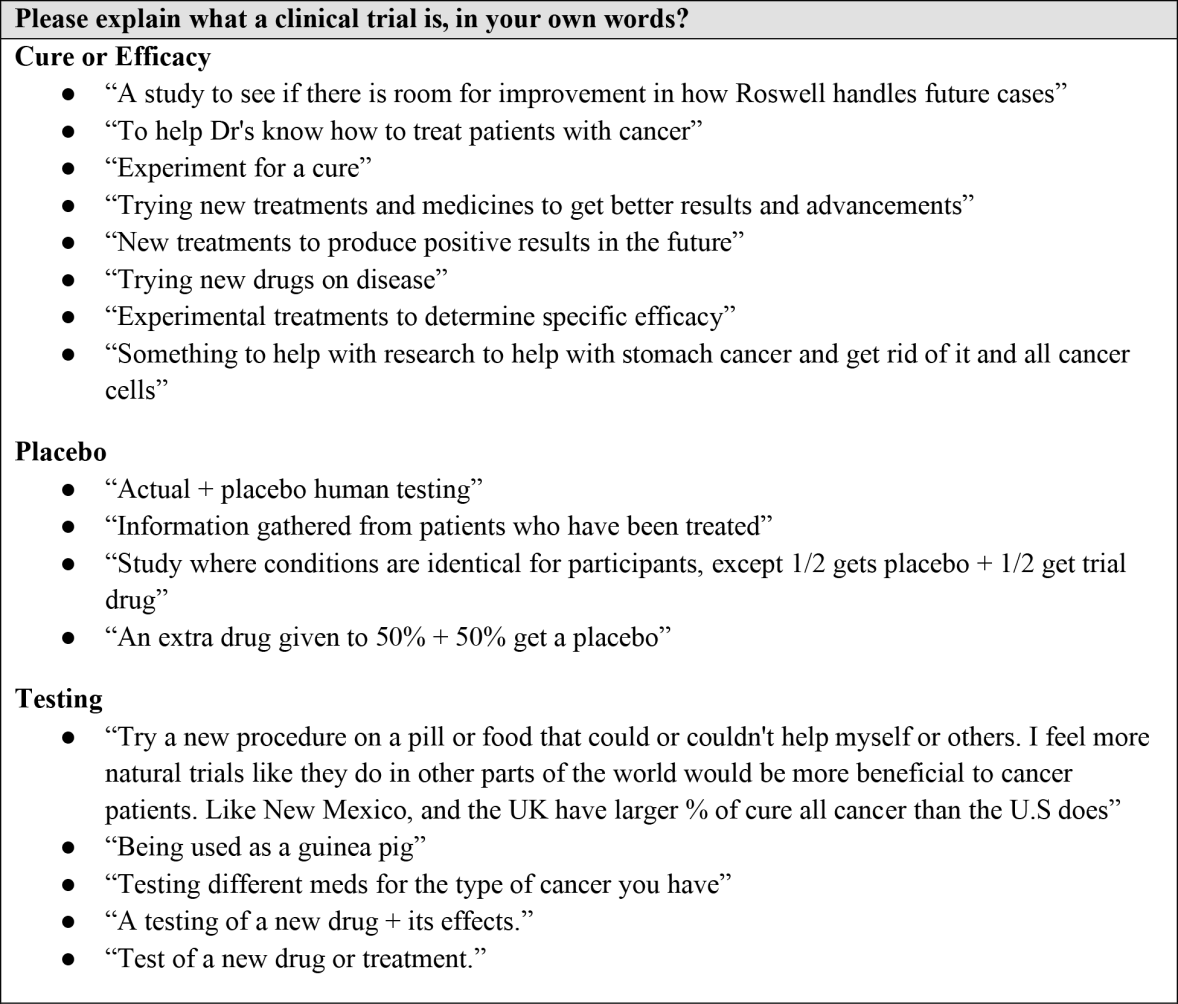
Write-In Responses

Figure 1 summarizes the participant topics, themes, and quotes.

## Discussion

We found that people who declined CT participation primarily cited concerns related to the ambiguity of effectiveness versus standard of care, the time required for participation in a trial, and lack of control of treatment choice.

Our findings support those in the literature regarding patient barriers to enrollment (Dias, Chao, Lee, Wu, & Kloecker, 2016; Hillyer et al., 2020; Lara et al., 2001; Manne et al., 2015; Mills et al., 2006; Network, 2021). The survey data suggest that those designing clinical trials should consider factors affecting the patient’s burden of participation. This has been examined extensively in the literature (Manne et al., 2015; Mills et al., 2006; Naidoo et al., 2020; Nielsen & Berthelsen, 2019; Sedrak et al., 2021). Making the frequency of clinic visits and time commitment equivalent to standard of care treatment should be considered a goal when designing trials. Our findings support that time can be a significant burden. When considering travel time only, 53% of the patients surveyed stated it took more than 30 mins to reach the cancer center and 29% reported that it took 1–2 hours.

While cost was not reported as a barrier by most respondents, indirect costs such as additional requirements for time (Nusbaum, Douglas, Damus, Paasche-Orlow, & Estrella-Luna, 2017) away from work or family care could result in a financial burden to patients (Nipp et al., 2019; Winkfield et al., 2018).

Wright and colleagues showed that perceived personal benefit was the most significant patient-related predictor of clinical trial enrollment (2004). Our analysis of write-in patient responses expands on this by providing additional perspective on the paradoxical concern about the risk of participation because, as one patient stated, it is “unknown if it would be better than standard of care”.

**Figure 2:**
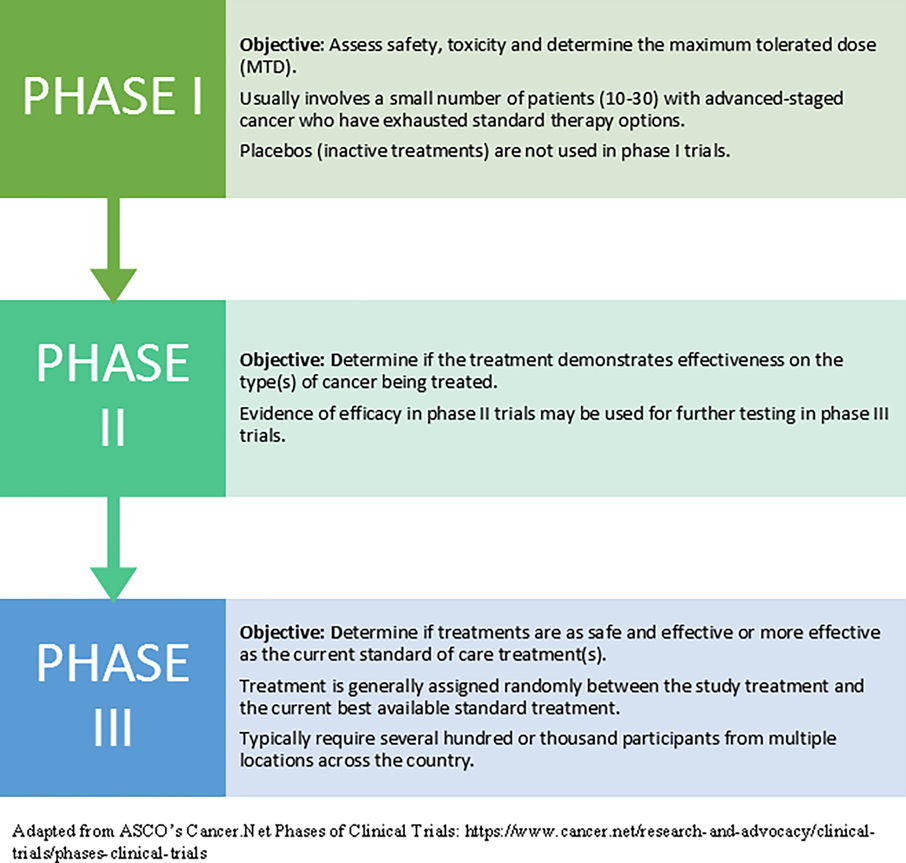
Phases of a Clinical Trial

Patient survey comments indicated confusion about the availability of efficacy data for the trial treatment offered. Some respondents did not appear to understand that efficacy data are not yet available for most phases of a clinical trial. For example, one patient stated they would be more willing to participate in a trial in the future by “knowing the results of testing in the past”. The provision of efficacy data to the patient depends on the phase of the trial offered. Refer to Figure 2: Phases of a Clinical Trial for a reference of clinical trial phases.

Patient-related barriers can be addressed through communication and education. Implementation of patient-level interventions such as the *Preparatory Education About Clinical Trials (PRE-ACT)* (Meropol et al., 2016) are useful in a prospective multi-site randomized clinical trial and should be considered for broad dissemination. The Meropol study is the largest randomized controlled trial to date that has looked at an intervention using a series of patient-facing educational videos specifically designed to address patient-level barriers. Topics address many of the barriers identified in this pilot study, including, “What is a placebo?”, “Will taking part in a clinical trial help me?”, and “Are there ways to deal with transportation and financial issues?”. Patients can watch this free series of educational videos on Cancer.Net, a patient information website managed by ASCO (Oncology, 2005–2020).

Ongoing work that addresses nursing interventions include an ongoing NIH-funded study, Oncology Nurse IMPACT: Improving Communication with Patients about Clinical Trial. IMPACT is testing the value of a tailored video-based educational intervention designed to increase oncology nurse intention to discuss clinical trials with patients. This study was built upon work by Flocke and colleagues that measured the attitudes, subjective norms and perceived behavioral control using survey data from over 1900 Oncology Nursing Society Members (2017).

A potential solution to the barrier of patient knowledge deficit suggested by Nipp and colleagues is the integration of patient navigators into the clinical trial accrual process (2019). Navigation has been shown to improve accrual to clinical trials in multiple studies, particularly to increase minority participation(Fouad et al., 2016; Rodrigues, Schneider, Kalinke, Kempfer, & Backes, 2021; Wells, Campbell, Kumar, Clark, & Jean-Pierre, 2018). Additionally, the *Education Network to Advance Cancer Clinical Trials* program recommends that documentation of prescreening of all patients for clinical trial eligibility and the inclusion of clinical trial navigators should be mandated (Nipp et al., 2019).

### Limitations

The project was conducted using a convenience sample at a single cancer center. Methodological limitations included a small survey sample size (n=21) that was 7% of the eligible patient population (n=272). Additionally, the study ratio of females to males was 2:1. Due to the small sample size, there were few subjects from minority groups. The patient survey, while used previously in a similar research context, was not a validated tool and was modified for the practice setting. Therefore, findings from the study may not be generalizable.

The use of the directed qualitative content analysis method to analyze the open-ended survey responses presents limitations. The directed approach can lead to confirmation bias, meaning that researchers are likely to find evidence that is supportive of a particular hypothesis (Hsieh & Shannon, 2005). There is also the potential that contextual features that may have influenced participant responses were not recognized. To reduce the amount of bias, study team members reviewed the data independently to confirm trustworthiness of the responses and published the complete set of write-in responses.

### Implications for Nursing

When addressing educational barriers, the entire healthcare team should help patients understand the purpose of clinical trials, and the potential value of trial participation. Nurses have many roles in their facilities and often have extended contact with patients, therefore, they are in a unique position to support patients in their decision regarding clinical trial participation. Nurses can provide targeted education, address patient identified concerns, and participate in the informed consent process. Understanding and assimilating themes identified in this study may enhance nurses’ ability to identify, teach and proactively discuss terms such as placebo, the idea of receiving “extra treatment”, and helping patients explore concerns about effectiveness of trial treatments.

Nurses should maintain proficiency through continuing education related to the design and importance of clinical trials. Nurses can also benefit from watching the patient video series *Preparatory Education About Clinical Trials (PRE-ACT)* to enhance their knowledge. The videos provide an example of how to present complex concepts such as “What is a placebo?” in a concise, understandable way to patients(Oncology, 2005–2020). Nurses can address concerns about availability of efficacy data for the trial treatment offered by reassuring patients that the study is usually testing current best therapy against something that may be better or less toxic.

Nurses may also be involved in providing informed consent. This role is recognized by the Oncology Nursing Society ([ONS], 2016) and the American Nurses Association ([ANA], 2016). The use of evidence-based nursing interventions such as teach-back to verify patient understanding during informed consent discussions is recommended (AHRQ, 2015). A key component of teach back is putting the responsibility of patient understanding on the nurse.

Studies such as *Clinical Trials Informed Consent: An educational intervention to improve nurses’ knowledge and communications skills* (Regan, 2018) have demonstrated the effectiveness of teach-back, an evidence-based health literacy intervention, during informed consent. This education intervention provides nurses with examples of teach-back scripts that can be used with patients in the informed consent process. The study demonstrated that after receiving teach-back training, nurses had high research knowledge scores and demonstrated statistically significant improvement in pre- and post-test conviction and confidence using teach-back(Regan, 2018).

### Conclusion

The results of this study can be used by all stakeholders to develop multifaceted interventions that include evidence-based education programs for nurses and patients and accommodations to support patients in minimizing the time and effort required to participate in a clinical trial. These findings also demonstrate key gaps in patient understanding of clinical trials and supports the need to conduct more extensive implementation studies on the feasibility and acceptability of evidence-based nursing interventions that have been shown to help address patient reported concerns about enrolling in clinical trials. Given their central role in oncology care, nurses should be considered integral members of the clinical research education program.

## Figures and Tables

**Table 1:** Demographics and Clinical Characteristics of Patients Surveyed

	N=21	
Age		
Range	41–69	
Mean	64	
Sex		
Male	7	33%
Female	14	67%
Race		
White	17	81%
African American	2	10%
American Indian/Alaskan Native	1	5%
Other or non SDecified	1	5%
Education		
High school	7	33%
Some College or no degree	3	14%
College	11	52%
Metastatic Status		
Metastatic	17	81%
Non Metastatic	4	19%
Tumor Types		
Breast	4	19%
Colorectal	4	19%
Gastrespohageal	3	14%
Non Small Celi Lung	3	14%
Ovarian	2	10%
Páncreas	3	14%
ProState	1	5%
Uterine	1	5%

**Table 2:** Trial Communication

Question	Yes	No
Did you feel you were given enough time to consider whether you wished to participate in the trial(s)?	n=20 (95%)	n=1 (5%)
Were you given the opportunity to ask questions before making your decision?	n=20 (95%)	n=1 (5%)
Would you have liked more time to ask questions?	n=3 (14%)	n=18 (86%)
Did you feel under pressure to participate in the trial that was offered?	n=2 (10%)	n=19 (90%)

**Table 3 –:** Patient Survey

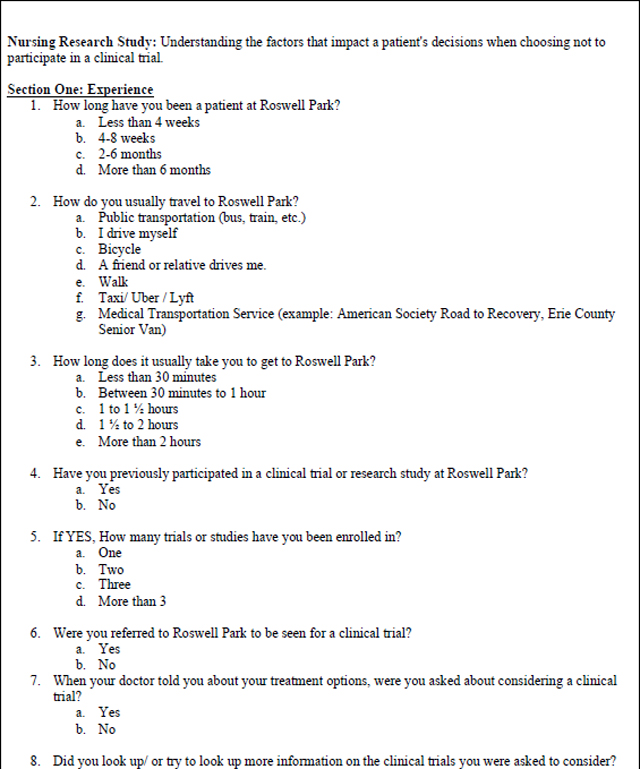
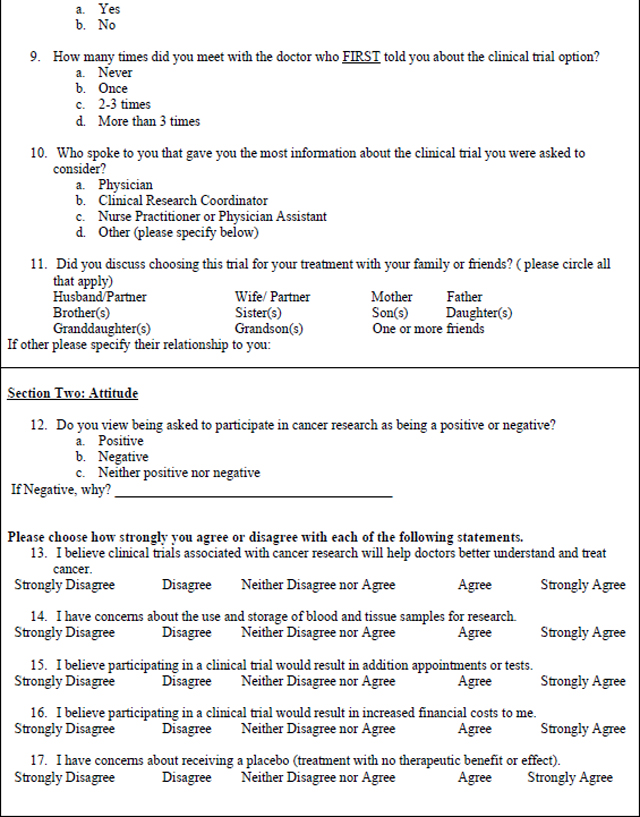
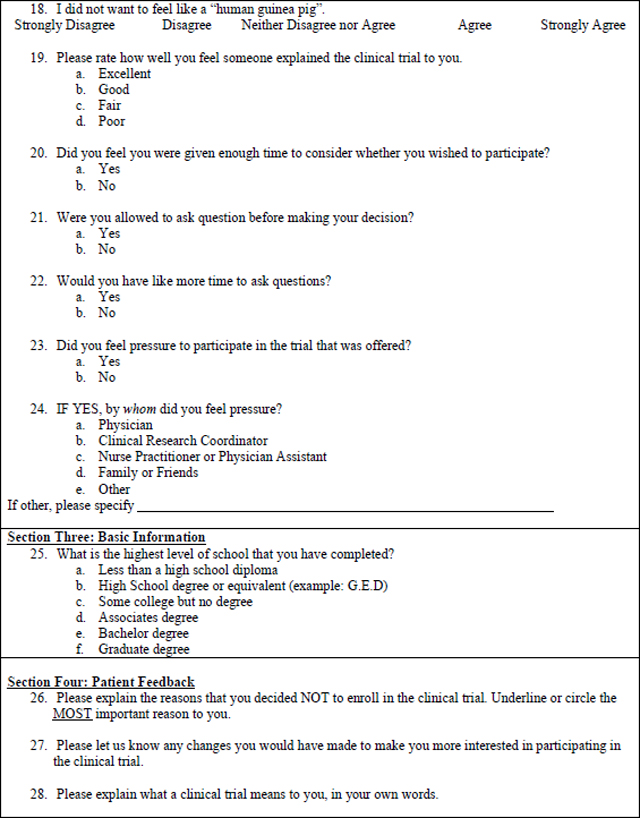

## Implications for Practice

- Proactively support and address the uncertainty patients feel about trial effectiveness when approached to participate in a clinical trial.
- Implement evidence-based, multifaceted interventions that include education programs for patients and nurses.
- Include the entire provider care team in helping patients understand the role and value of clinical trials.
